# Identification on surrogating overall survival with progression-free survival of first-line immunochemotherapy in advanced esophageal squamous cell carcinoma—an exploration of surrogate endpoint

**DOI:** 10.1186/s12885-023-10613-y

**Published:** 2023-02-10

**Authors:** Zewei Zhang, Chunxia Xie, Tiantian Gao, Yuxian Yang, Yong Yang, Lei Zhao

**Affiliations:** 1 Department of Radiation Oncology, Sun Yat-Sen University Cancer Center, Guangzhou, China; 2 State Key Laboratory of Oncology in South China, Guangzhou, China; 3 Collaborative Innovation Center for Cancer Medicine, Guangzhou, China; 4 Department of Radiation Oncology, Fujian Medical University Union Hospital, Fuzhou, China

**Keywords:** Surrogate, Progression-free survival, PD-1/PD-L1 inhibitors, Advanced esophageal squamous cell carcinoma

## Abstract

**Background:**

Overall survival (OS) is the gold standard to assess novel therapeutics to treat cancer. However, to identify early efficacy and speed up drug approval, trials have used progression-free survival (PFS) as a surrogate endpoint (SE). Herein, we aimed to examine if PFS could function as an OS surrogate in advanced Esophageal Squamous Cell Carcinoma (ESCC) treated with first-line immunochemotherapy.

**Methods:**

Two hundred ninety-two advanced ESCC patients treated using inhibitors of PD-1/PD-L1 + chemotherapy or chemotherapy alone were collected. In addition, six phase III randomized clinical trials were eligible for inclusion. Bayesian normal-induced-copula-estimation model in retrospective patient data and regression analysis in the published trial data were used to determine the PFS-OS correlation.

**Results:**

PFS correlated moderately with OS in the retrospective cohort (Kendall’s Tau = 0.684, τ = 0.436). In trial-level, treatments effects for PFS correlated weakly with those for OS in intention-to-treat population (R^2^ = 0.436, adj.R^2^ = 0.249, *P* > 0.05) and in PD-L1-enriched population (R^2^ = 0.072). In arm-level, median PFS also correlated weakly with median OS. Moreover, analysis of the retrospective cohort demonstrated that the annual death risk after progression in the continued immunotherapy group was considerably lower than that in the discontinued group.

**Conclusion:**

In trials of anti-PD-1 agents to treat advanced ESCC, the current results provide only weak support for PFS as an OS surrogate; OS cannot be substituted completely by PFS in these cases. The results also suggest that qualified patients with advanced ESCC might benefit from continuous immunotherapy beyond progression to achieve a decreased risk of death.

**Supplementary Information:**

The online version contains supplementary material available at 10.1186/s12885-023-10613-y.

## Background

More than 600,000 people worldwide are diagnosed with esophageal cancer (EC) every year, and 5.5% of all patients with malignant tumors die of EC, ranking sixth among global cancer death rate [1]. In China, histologically, over 90% of EC is esophageal squamous cell carcinoma (ESCC) [2]. At present, platinum-containing doublets chemotherapy is the standard first-line (1-L) treatment of metastatic or advanced ESCC. Unfortunately, chemotherapy alone can lead to poor survival, equating to median overall survival (OS) of 7–13 months as well as a less than 20% 5-year OS [3, 4]. Recently, it has been reported that, compared with chemotherapy alone, the combination of programmed death 1 (PD-1) inhibitors with chemotherapy has clinical benefits [5, 6]. PD-1/PD-L1 inhibitors employ unique mechanisms to activate autoimmunity to tumors, which might lead to a long duration of response, responses after discontinuation of therapy, and long-term survival benefits [7–9]. Therefore, it is doubtful whether the trial end points based on the traditional RECIST criteria, such as PFS, can capture these atypical response patterns [10–12].

OS is the gold standard end point to assess novel therapeutics to treat cancer. However, to conduct clinical trials faster and possibly speed up drug approval, progression-free survival (PFS) is frequently employed as an alternative endpoint of OS [13–15]. However, meta-analyses of immunotherapy-based trials revealed that the PFS-assessed treatment effect correlated only poorly or moderately with OS, and the ongoing use of PFS as a surrogate endpoint (SE) in the immunotherapy era was questioned [16, 17]. In addition, the long-term efficacy of immunotherapy means the post-progression survival (PPS) tended to improve and treatment beyond progression (TBP) could confer a survival benefit in patients treated using inhibitors of PD-1/PD-L1 [18–20]. Herein, we aimed to determine if PFS can be employed as an OS surrogate in advanced ESCC patients treated using first-line immunochemotherapy, and to study PPS and the risk of death after progressive disease (PD) in patients who received immunochemotherapy, aiming to guide future PD-1 research and clinical practice.

## Materials and methods

### Searching the literature and selecting studies

Comprehensive searches of the literature in the Cochrane, Embase and PubMed databases were performed to identify articles published prior to May 1, 2022. The search used the following keywords: (“randomized controlled trial” OR “controlled clinical trial”) AND (“Nivolumab” OR “Atezolizumab” OR “Tislelizumab” OR “Sintilimab” OR “Toripalimab” OR “cemiplimab” OR “Durvalumab” OR “Pembrolizumab” OR “Camrelizumab” OR “Avelumab” OR “Lambrolizumab” OR “BMS-936558” OR “MDX1106” OR “ONO-4538” OR “MDX1105” OR “MPDL3280” OR “MPDL3280A” OR “RG7446” OR “MEDI4736” OR “REGN2810” OR “SHR-1210” OR “JS001” OR “immune checkpoint inhibitor” OR “ICI” OR “immunotherapy” OR “PD-1”) AND (“esophageal” OR “oesophageal” OR “Gastroesophageal”) AND (“cancer” OR “carcinoma” OR “tumor” OR “neoplasm”). The eligibility criteria included randomized clinical trials (RCTs) dealing with the long-term survival of patients suffering from previously untreated, locally advanced, unresectable or metastatic ESCC who were treated using PD-1/PD-L1 inhibitors plus chemotherapy or with chemotherapy alone. We excluded the clinical trials designed for second line or later therapy, and trials with neoadjuvant or adjuvant settings. We also excluded single-arm phase I or phase II trials.

**Fig. 1:**
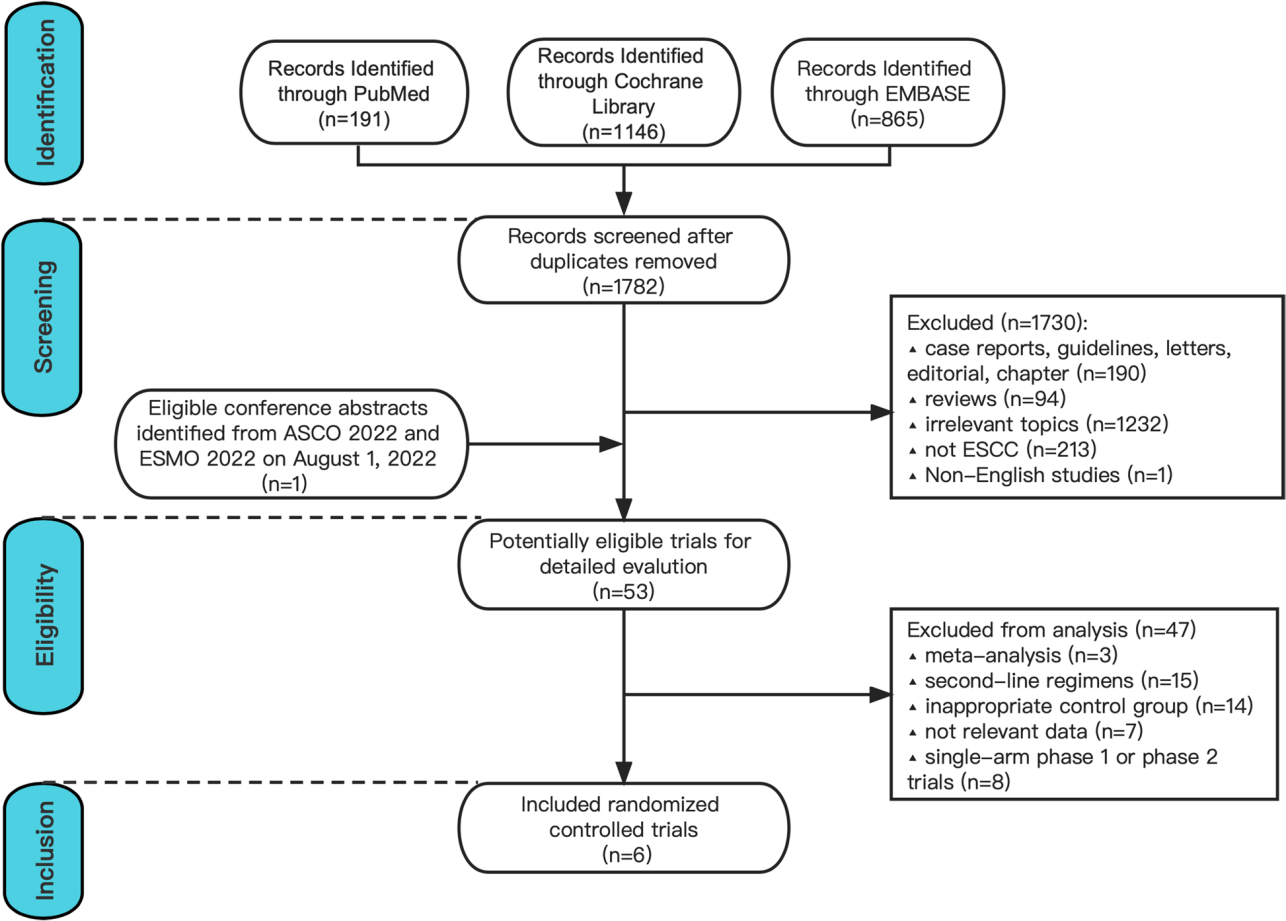
Flow chart of the study selection. Phase II and III RCT PRIMA flowchart. Abbreviations: RCTs, randomized controlled trials

Published trial reports from January 1, 1975 to May 1, 2022 were searched for in PubMed, EMBASE, and the Cochrane Library. The online supplemental methods (Supplemental Methods) show the detailed search strategy. After excluding duplicates, 1782 references were screened, and review of the title and abstract resulted in 1730 being disregarded (Fig. 1). Trials eligible for inclusion were searched manually by searching conference abstracts of 2022 American Society of Clinical Oncology (ASCO) annual meeting as well as 2022 European Society for Medical Oncology (ESMO) Congress. After reading the full text, among the 53 potentially eligible articles, only 6 studies (phase III RCTs) were included for trial-level analyses [5, 6, 21], including JUPITER-06 [5], CHECKMATE-648 [6], ESCORT-1st [21], ORIENT-15 [22], KEYNOTE-590 [23] and RATIONALE-306 [24]. Two of the studies were carried out in China and four were carried out in other countries. The characteristics and outcomes of the six included RCTs are shown in Supplemental Table 1. Since our research focus is on the ESCC population taking PD-1/PD-L1 inhibitors in conjunction with chemotherapy as well as ESCC population receiving chemotherapy alone, we did not include the results of the nivolumab plus iplimumab arm in CHECKMATE-648 trial, and excluded the data of adenocarcinoma patients in KEYNOTE-590 trial. The RCTs reported data for 3611 trial participants, 1803 of whom were treated using agents that block PD-1 and PD-L1 combined with chemotherapy for ESCC, and 1808 received chemotherapy plus placebo. In order to determine the expression level of PD-L1, CHECKMATE-648 applied the PD-L1 immunohistochemical assay 28–8, KEYNOTE-590 and ORIENT-15 applied the assay 22C3, ESCORT-1st applied the assay 6E8, JUPITER-06 applied the assay JS311, and RATIONALE-306 applied the assay SP263. An evaluation of heterogeneity across studies was conducted using the Chi-square test (Heterogeneity is defined as *P* < 0.1 and non-heterogeneity as *P* > 0.1) and the I^2^ statistic (I^2^ > 50% indicates substantial heterogeneity). Using Egger's test, significant publication bias was noted when *P* >|t|< 0.05 was determined. Heterogeneity was not observed (Supplement Fig. S1), nor did the Egger's publication bias plot show evidence of publication bias across studies (Supplement Fig. S2). The eligibility of relevant articles was determined by retrieving and assessing the full texts. Two authors (Zhang ZW and Gao TT) performed the literature search independently. Any discrepancies were resolved by consensus.

**Table 1:** Baseline characteristics of patients who received chemotherapy in combination with immunotherapy or chemotherapy alone

Characteristics	Chemotherapy + PD-1/L1	Chemotherapy alone	*P* value
Number	190	102	
Gender, n (%)			0.728
Male	163 (85.8%)	89 (87.3%)	
Female	27 (14.2%)	13 (12.7%)	
Age(years), mean ± sd	60.437 ± 7.8359	61.99 ± 11.686	0.230
Baseline ECOG PS, n (%)			0.201
1	140 (73.7%)	82 (80.4%)	
0	50 (26.3%)	20 (19.6%)	
Disease status at trial entry, n (%)			< 0.001
Metastatic	77 (40.5%)	63 (61.8%)	
Unresectable advanced	46 (24.2%)	31 (30.4%)	
Recurrent, distant	38 (20%)	3 (2.9%)	
Recurrent, locoregional	29 (15.3%)	5 (4.9%)	
Hepatic Metastases, n (%)			0.046
NO	163 (85.8%)	78 (76.5%)	
YES	27 (14.2%)	24 (23.5%)	
Pulmonary Metastases, n (%)			0.316
NO	149 (78.4%)	85 (83.3%)	
YES	41 (21.6%)	17 (16.7%)	
Bone Metastases, n (%)			0.143
NO	180 (94.7%)	92 (90.2%)	
YES	10 (5.3%)	10 (9.8%)	
Site, n (%)			0.061
Lower	61 (32.1%)	38 (37.3%)	
Middle	67 (35.3%)	36 (35.3%)	
Cervical	10 (5.3%)	2 (2%)	
Upper	36 (18.9%)	10 (9.8%)	
Multiple	16 (8.4%)	16 (15.7%)	
Baseline clinical stage (AJCC 8th), n (%)			0.223
IV	164 (86.3%)	93 (91.2%)	
III	26 (13.7%)	9 (8.8%)	
Histological grade, n (%)			0.267
Well or moderately differentiated	97 (51.1%)	59 (57.8%)	
Poorly differentiated or undifferentiated	93 (48.9%)	43 (42.2%)	
Chemotherapy regimen of the study treatment, n (%)			0.117
Platinum + Fluorouracil	46 (24.2%)	27 (26.5%)	
Platinum + Paclitaxel	120 (63.2%)	68 (66.7%)	
Fluorouracil + Irinotecan	7 (3.7%)	1 (1%)	
Irinotecan	4 (2.1%)	0 (0%)	
Fluorouracil	6 (3.2%)	0 (0%)	
Paclitaxel	5 (2.6%)	2 (2%)	
Others	2 (1.1%)	4 (3.9%)	
Chemotherapy in postprogression stage, n (%)			0.357
Paclitaxel	8 (21.6%)	2 (10.5%)	
Fluorouracil + Irinotecan	17 (45.9%)	10 (52.6%)	
Platinum + Paclitaxel	2 (5.4%)	0 (0%)	
Irinotecan	5 (13.5%)	4 (21.1%)	
Fluorouracil	2 (5.4%)	3 (15.8%)	
Others	3 (8.1%)	0 (0%)	
Continuing immunotherapy or immunotherapy in postprogression stage, n (%)			0.452
Toripolimab	9 (25.7%)	4 (44.4%)	
Sintilimab	17 (48.6%)	2 (22.2%)	
Camrelizumab	4 (11.4%)	2 (22.2%)	
Nivolumab	3 (8.6%)	0 (0%)	
Tislelizumab	1 (2.9%)	1 (11.1%)	
Pembrolizumab	1 (2.9%)	0 (0%)	

Abbreviations: *IQR* interquartile range, *ECOG* Eastern Cooperative Oncology Group, *PS* performance status, *AJCC* American Joint Committee on Cancer

### Criteria to select individual patients

Patients diagnosed ESCC between 2018 and 2021 at the Sun Yat-sen University Cancer Center were retrospectively reviewed. Inclusion of patients was appropriate if they were at least 18 years and had ESCC that was locally advanced, recurring, or metastatic (regardless of PD-L1 status) on histology or cytology, and measurable disease per RECIST version 1.1, and a performance status of 0–1 for the Eastern Cooperative Oncology Group, and hematological and organ function were adequate; they were not amenable to radical surgery or definitive chemoradiotherapy; and they had not been treated previously with systemic anticancer agents (patients who experienced progression > 6 months after chemoradiotherapy or surgery were eligible which referred to ESCORT-1st [21], JUPITER-06 [5], ORIENT-15 [22]). The following criteria were excluded: presence of other malignancies; central nervous system metastases; active or previous autoimmune or inflammatory diseases; prior use of immune checkpoint blockade, systemic immune stimulation, and/or systemic immunosuppression. The inclusion and exclusion criteria of patients referd to six phase III randomized clinical trials, including ESCORT-1st, JUPITER-06, ORIENT-15, KEYNOTE-590, CHECKMATE-648 and RATIONALE-306.

### Statistical analyses

We defined OS as the interval between the start of randomization or treatment and death from any cause. We defined investigator-assessed PFS as the period between the start of randomization or therapy and clinical or radiological progression, or death. As defined by the PPS, it was the period from progression to death from any cause. The estimated hazard ratios (HRs) of progression and death overtime were smoothened using the Epanechnikov kernel. A Kaplan–Meier survival curve was used in comparison of two groups in terms of time-to-event. A valid surrogate for OS can be found at an early end point, such as PFS, if the efficacy of treatment on OS is totally captured by that on the early end point (also known as "Prentice" criteria) [25]. Despite the difficulty of directly measuring Prentice criteria, good surrogates must exhibit strong correlations to an endpoint in reality (associations at the patient level) and a strong correlation is needed between effects of treatment on surrogates and those on the real endpoint (associations at the trial level) [26]. In order to determine whether PFS is a SE for OS, the association between the endpoint and OS must be evaluated at both the patient and trial levels [27]. For the analysis in the retrospective patient cohort, PFS and OS correlations were assessed with an estimation model based on Bayesian normal induced copulas [28], which accounts for censoring time-to-event endpoints such as OS and PFS. PFS and OS duration (months) were measured for their relevance using Kendall’s Tau, in which longer PFS is correlated with longer OS when the positive value is very close to 1.

We examined the correlations between PFS and OS HRs according to the results available in patients with intention-to-treat (ITT) and populations with PD-L1 enrichment (based on literature search data) for treatment effect (trial-level analyses). Supplement Table 2 shows the characteristics and outcomes of RCTs included in analyses of PD-L1-positive population. We analyzed PD-L1 subpopulations reported in the literature (PFS data of PD-L1 subgroups in the RATIONALE-306 study has not been reported in the abstract at the ESMO 2022 Annual Meeting [24]); PD-L1-positive populations assigned to populations for primary or secondary analyses were utilized for trials that studied several subgroups of PD-L1. A definition of positive for PD-L1 is combined positive score (CPS) ≥ 10 in KEYNOTE-590 and ORIENT-15, CPS ≥ 10% in RATIONALE-306, CPS ≥ 1 in JUPITER-06, and tumor proportion score (TPS) ≥ 1% in ESCORT-1st and CHECKMATE-648. At the level of clinical trials, in order to quantify the correlation between treatment effects [log hazard ratios (HRs)] on OS and PFS, a linear regression model was used with weighted Pearson correlation coefficient (R), weighted by trial size. To assess the proportion of the variation explained by the surrogate, at the trial level, we calculated the squared correlation coefficient R^2^. If both Kendall’s Tau (patient level association) and R^2^ (trial level association) were close to 1.00, only then were the candidate surrogate end points considered acceptable [26, 27]. Based on this regression model fit, if the R^2^ estimate was less than 0.4, PFS was not considered appropriate for explaining OS viability using the model, and this model was not evaluated further [29–31].

However, in terms of statistical evaluation criteria for biomarker-surrogacy validity, a retrospective cohort study evaluating marker-target relationships ranks 0 as having a very poor validity [32]. In order to increase reliability of assessing PFS as a potential surrogate endpoint for OS, another correlation approach (arm-level) was used according to previous descriptions [26, 33]. A quantitative assessment of the association between median PFS and median OS across immunochemotherapy -treated experimental arms was conducted. The arm-level correlation was also quantified by Pearson correlation coefficient (R) via a linear regression model with trial size weighting. In accordance with the Institute for Quality and Efficiency in Health Care (IQWIG) criteria, a strong association between endpoints was defined as R greater than 0.85, moderate (R = 0.70–0.85) or weak (R lower than 0.70) [34]. Statistical analyses were conducted using SPSS version 24.0, R version 4.04 and Stata/SE 17.0. The significance of the data was determined by a two-sided P of less than 0.05.

## Results

### Characteristics of the patients at baseline and their treatment

Table 1 showed a description of the patients' clinical characteristics at baseline. There is almost no significant statistical difference in baseline characteristics between the two groups. Mean age of 60.437 and 61.99 years were observed in patients who received chemotherapy in combination with immunotherapy or chemotherapy alone, respectively; 6.3:1 was the ratio of male to female. Most of the patients had an Eastern Cooperative Oncology Group performance status (ECOG PS) score of 1 (76%) as well as stage IV (88%) disease. The disease status of patients at trial entry included metastatic (*n* = 140, 47.9%), unresectable advanced (*n* = 77, 26.4%) and recurrent (*n* = 75, 25.7%). There were a median of 6 cycles of chemotherapy in both groups and median number of cycles of anti-PD-1/PD-L1 therapy received by the trial group was six. The most frequently used chemotherapy regimen was platinum + fluorouracil or platinum + paclitaxel (*n* = 261, 89.4%). The most frequently used anti-PD-1/PD-L1 treatments in the Chemotherapy + PD-1/L1 group were toripolimab (*n* = 60, 31.6%), sintilimab (*n* = 67, 35.3%), or camrelizumab (*n* = 45, 23.7%). In the three treatment regimens, including chemotherapy regimen of the study treatment, subsequent chemotherapy in postprogression stage and continuing immunotherapy in postprogression stage, there is no statistical difference between Chemotherapy + PD-1/L1 group and chemotherapy alone group.

### Comparison of PFS and OS and the Annual hazard ratio in the retrospective cohort of patients

**Fig. 2:**
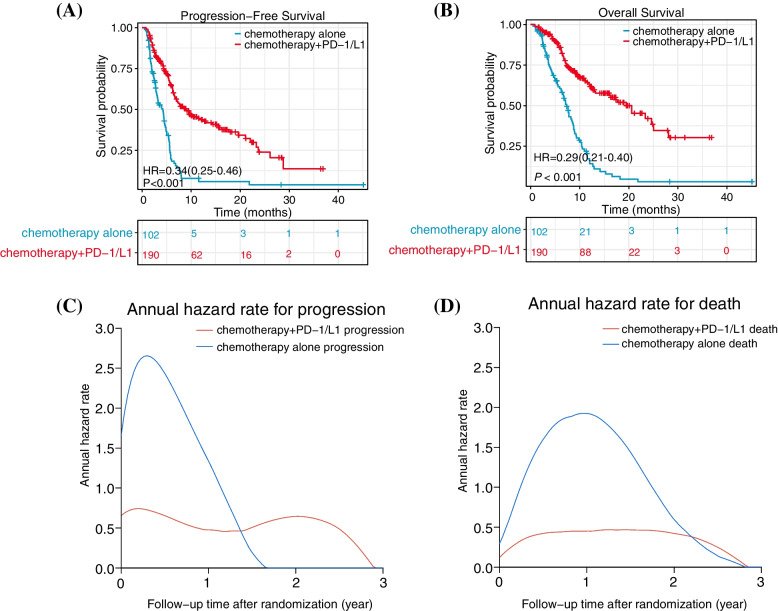
Whole cohort estimates of PFS and OS and the annual hazard rate over time. **A** 75 of 190 (39.5%) patients in the P + C group and 78 of 102 (76.5%) patients in the CA group died. **B** 108 of 190 (56.8%) patients in the P + C group and 81 of 102 (79.4%) in the CA group experienced PD or death. **C** Whole cohort annual hazard rate for progression. **D** Whole cohort annual hazard rate for death. Abbreviations: OS, overall survival; PD-1/L1, agents that inhibit PD-1 or PD-L1; PFS, progression-free survival; HR, hazard ratio

At the time of data cutoff (March 18,2022), a median of 14.8 months (interquartile range [IQR], 12.23–16.7 months) have been followed up. 108 of 190 patients (56.8%) in the PD-1/PD-L1 + chemotherapy (P + C) group and 81 of 102 patients (79.4%) in the chemotherapy alone (CA) group had experienced progressive disease (PD) or death; 75 (39.5%) patients in the P + C group and 78 (76.5%) in the CA group died. Compared with chemotherapy only, immunochemotherapy improved OS significantly (median OS 20.6 *versus* 7.3 months; HR = 0.29; 95% CI: 0.21–0.40) (Fig. 2A). PFS was obviously improved in the P + C group in comparison to that in the CA group (median PFS 9 *vs*. 4.2 month; HR = 0.34; 95% CI: 0.25–0.46) (Fig. 2B). Consistently, according to the smoothed hazard plot (Fig. 2C), in the CA group, the risk of progression was higher and decreased to zero earlier than in the P + C group. The highest annual progression hazard was 2.67 *vs.* 0.74, and times of hazard reduction to 0 were 1.66 *vs.* 2.88 years. Figure 2D shows that the risk of death in the CA group had a higher percentage than that in the P + C group. The highest annual death hazard was 1.92 *vs.* 0.47, and times of hazards reduction to 0 were both 2.79 years. However, whether for progression or death, the trend of the annual hazard rate in the P + C group was significantly different from that in the CA group. Unlike the significant single peak for the CA group, the risk change of the P + C group was gentle and had a long plateau period.

### Retrospective cohort analyses: correlations between OS and PFS in patients treated using inhibitors of PD-1/PD-L1 plus chemotherapy

**Fig. 3:**
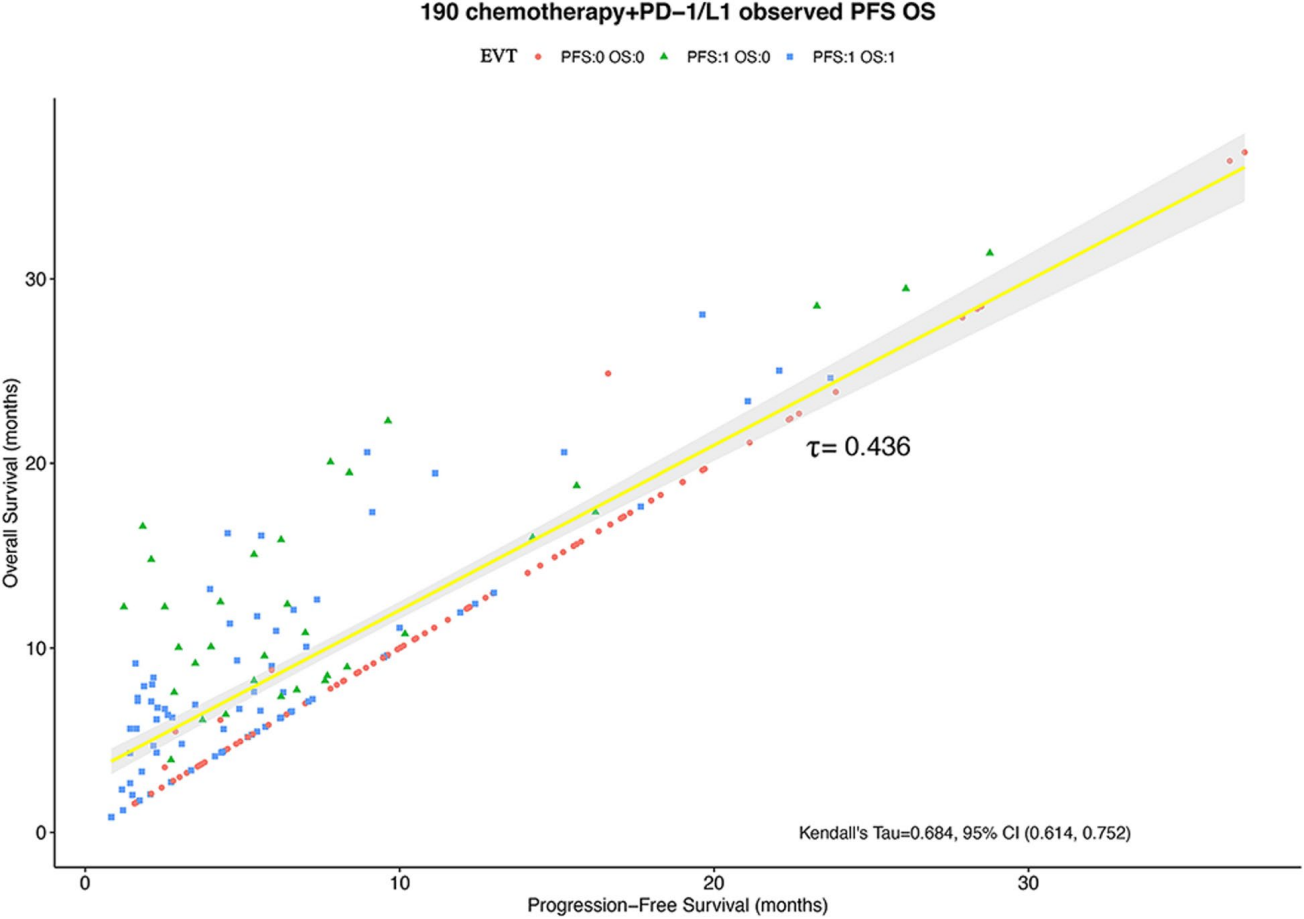
Correlation between OS and PFS in advanced ESCC patients treated using immunochemotherapy. The patient experienced no event during follow-up, including disease progression or death, indicated by a "0". A "1" indicates that the event of interest took place. Abbreviations: EVT, event; PD-1/L1, anti-PD-1 or anti-PD-L1 agents; CI, confidence interval; OS, overall survival; PFS, progression-free survival

We identified a duration of PFS is positively associated with OS in 190 immunochemotherapy-treated patients with advanced ESCC (Kendall’s Tau = 0.684; 95%CI: 0.614–0.752; Fig. 3). Considering the time-to-event endpoints are subject to censorship, we re-calculated Kendall’s Tau with an estimation model based on Bayesian normal induced copulas [28], giving a τ value of 0.436. Notably, despite a modest PFS duration, prolonged OS may still occur in the retrospective cohort of patients.

### Trial- and arm- level exploration of the PFS-OS correlation

**Fig. 4:**
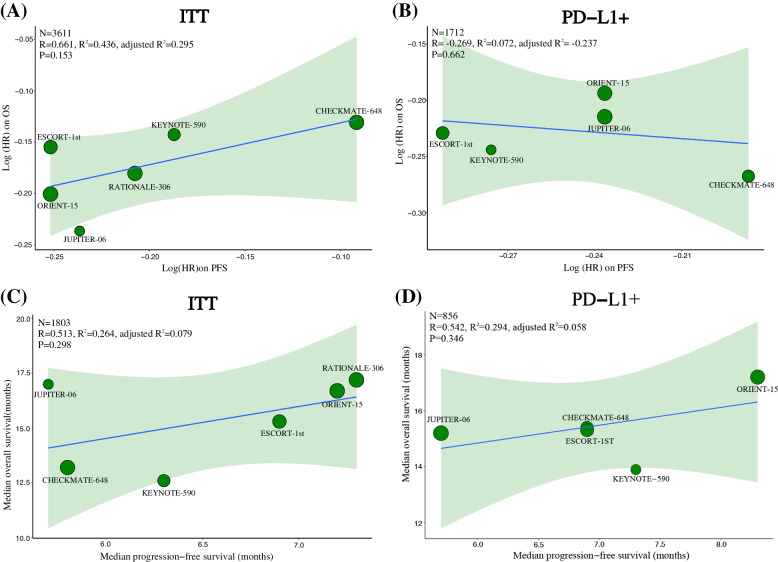
PFS-OS correlation at the trial and arm levels. **A**-**B** Correlation between the HR_OS_ and HR_PFS_ in ITT and PD-L1 + population at trial- level. **C**-**D** Relationship between median PFS and median OS in ITT and PD-L1 + population at arm- level. In each comparison, the number of patients is proportional to the size of the circle. Blue shows a weighted linear regression line and green shows its 95% CI. *n* indicates total number of patients enrolled in RCTs or experimental arms (arms treated with immunochemotherapy). R stands for weighted Pearson correlation coefficient. *R*^*2*^ is the squared correlation coefficients. Abbreviations: CI, confidence interval; HR, hazard ratio; ITT, intent-to-treat; PD-L1 + , programmed death ligand-1 enriched; RCTs, randomized controlled trials; OS, overall survival; PFS, progression-free survival

In the trial data, there was a weak correlation of the treatment effects on PFS with treatment effects on OS in the ITT population (R = 0.661, R^2^ = 0.436) and PD-L1 positive population (R = -0.269, R^2^ = 0.072) calculated using log HRs from Cox models (Fig. 4A, B). Thus, only 43.6% and 7.2% of the variation in weighted efficacy of treatment on OS was described by efficacy on PFS for the ITT and PD-L1 positive populations, respectively. In arm-level, there were 6 available experimental arms in ITT and 5 in PD-L1 + population included to analysis the correlation between the possible endpoint and OS. It was found that median PFS (mPFS) and median OS (mOS) were both weakly correlated in the ITT (R = 0.513, *P* = 0.298, Fig. 4C) and PD-L1-positive populations (R = 0.542, *P* = 0.346, Fig. 4D). As a result of the weighted regression, the adjusted R^2^ was only below 0.4 and *P* value > 0.05 for both the two populations in trial- and arm- level; therefore, the goodness-of-fit for a linear regression was poor and a positive linear correlation was not observed between effects of treatment on PFS and those on OS, and nor was there any positive linear correlation between mPFS and mOS. So, further predictions were not made using this regression model.

### Post-progression survival in the retrospective patients cohort

**Fig. 5:**
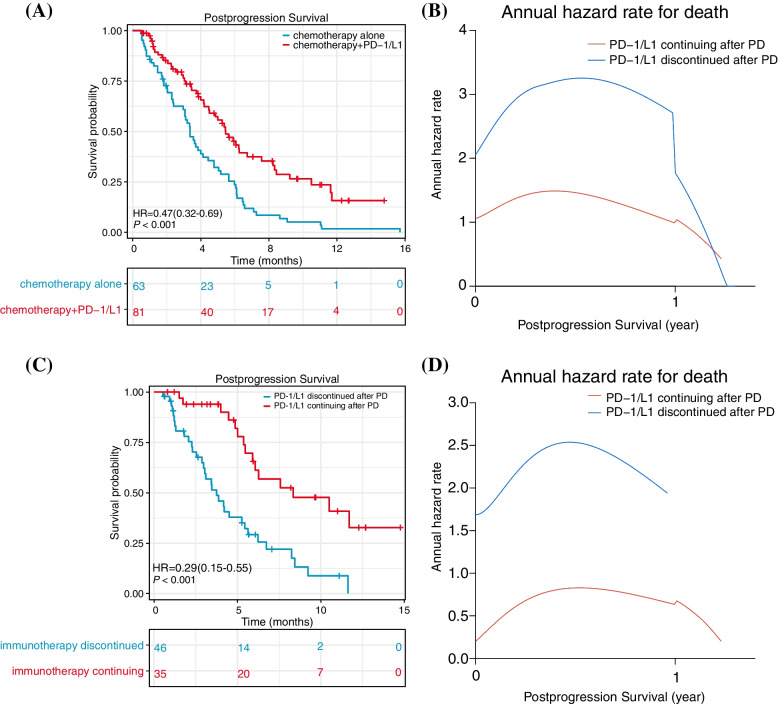
Estimates of Post-progression Survival and the Annual hazard rate for death after progression. **A** 48 of 81 (59.3%) patients in the P + C group and 60 of 63 (95.2%) patients in the CA group died. **B** Annual hazard rate for death after PD. **C** 15 of 35 (42.9%) patients who continued with PD-1/PD-L1 inhibitors after PD and 33 of 46 (71.7%) patients who discontinued PD-1/PD-L1 inhibitors died. **D** Annual hazard rate for death after PD in the P + C group. Abbreviations: PD-1/L1, anti-PD-1or anti-PD-L1 agents; PD, progressive disease; HR, hazard ratio

We analyzed the PPS of 144 enrolled patients who were judged to have disease progression. The PPS of the P + C Group lengthened significantly in comparison with that of the CA group (median PPS = 5.5 *vs.* 3.4 months; HR = 0.47; 95% CI 0.32–0.69) (Fig. 5A). In addition, after being judged as disease progression, the annual death risk of the P + C Group was considerably lower than that of CA group at the same time (Fig. 5B). The peak hazard rate of death was 1.49 *vs.* 3.25. After that, we continued to study the PPS of patients who were judged as having PD in the P + C Group, and divided them into two groups according to whether they continued to use inhibitors of PD1/PD-L1. The results showed that the PPS of patients who continued to use PD-1/PD-L1 inhibitors after PD was considerably longer compared with those who stopped using the drugs (median PPS = 8.3 *versus* 3.7 months; HR = 0.29; 95% CI, 0.15–0.55) (Fig. 5C). The annual death risk of patients who continued immunotherapy was significantly lower than that of those who discontinued (Fig. 5D). The peak hazard rate of death was 0.83 *vs.* 2.54.

## Discussion

As far as we know, this was the first study to identify PFS-based endpoints systematically in advanced ESCC patients receiving immunotherapy in combination with chemotherapy as 1-L treatment. Previous studies have shown that three-year PFS is a reliable SE of 5-year OS in locally advanced ESCC treated with definitive RT; when treating resectable ESCC and esophageal adenocarcinoma (EAC) and gastroesophageal junction (GEJ) cancer, HR_PFS_ can be used in neoadjuvant, perioperative, or adjuvant settings as a substitute for HR_OS_; in addition, in neoadjuvant RCTs of gastric carcinoma(GC) and GEJ adenocarcinoma, event-free survival (EFS) could serve as a SE [35–37]. However, in the immune-oncology (IO) era, due to the special response patterns of Immuno-checkpoint inhibitors (ICIs) such as pseudoprogression and delayed response, whether the traditional SE for OS is applicable to IO trials is controversial [38–40]. In 2018, a meta-analysis of trial data investigating immunotherapies in different solid tumors and different treatment lines revealed that PFS and OS had a weak correlation, according to Mushti et al. [16]. Yet, a recent study reported that when evaluating immunotherapy clinical trials for gastro-esophageal cancer, PFS may serve as a suitable surrogate for OS. Notably, heterogeneity in that study cannot be ruled out, including heterogeneity in cancer types (GC, GEJ adenocarcinoma, EAC and ESCC) as well as lines of treatment (1-L, ≥ 2 lines) and treatment strategies (Monotherapy and Combinational therapy). Therefore, it is necessary to verify whether PFS is a valid SE in clinical trials of 1-L immunotherapy for advanced ESCC. In this study, we evaluated the PFS-OS correlation in a retrospective cohort of patients, and a widely-accepted meta-analytic two-stage validation model was used to identify whether PFS can serve as a SE for OS in the ITT and PD-L1-enriched groups at the trial-/arm-level. However, PFS was revealed as an invalid surrogate for OS in advanced ESCC treated immunochemotherapy as first-line treatment (patient level τ = 0.436, trial level squared correlation R^2^ = 0.436 in ITT and R^2^ = 0.072 in PD-L1 + , arm level R^2^ = 0.264 in ITT and R^2^ = 0.294 in PD-L1 +). In addition, we found inhibitors of PD-1/PD-L1 associated with a relatively long PPS in the retrospective cohort, which induced a dilution effect in detecting the treatment effect on OS derived from PFS [41].

At the patient level of retrospective data, we found that the annual hazard rates of disease progression and death decreased in P + C group compared with those in the CA group. Unlike the unimodal distribution of chemotherapy alone, the risk variation of PD or death in immunotherapy combination treatment is mild and has a long plateau period. PPS also lengthened and the annual risk of death after PD was reduced. This could be explained by the long duration of remission and delayed effect of immunotherapy [7, 8]. Response patterns to immunotherapy may differ from traditional chemotherapy [42]. A longer PPS may also be related to pseudoprogression [43], which refers to the phenomenon that the tumor first increases in size because of T-cell infiltration prior to shrinkage. In this case, investigators might consider the response to be progressive disease under RECIST V1.1. As a result of the patient responding later, the patient's overall survival is improved. At the patient level, PFS correlated moderately with OS (Kendall’s Tau = 0.684, 95% CI 0.614–0.752, τ = 0.436); at the arm level, mPFS weakly correlated with mOS in ITT (R^2^ = 0.264, adj.R^2^ = 0.079, *p* = 0.298) and PD-L1-enriched populations (R^2^ = 0.294, adj.R^2^ = 0.058, P = 0.346); at the trial level, treatments effects (logHRs) on PFS also correlated weakly and even inversely with those on OS in ITT (R^2^ = 0.436, adj.R^2^ = 0.249, *P* = 0.295) and PD-L1-enriched populations (R^2^ = 0.072, adj.R^2^ = -0.237, *P* = 0.662). In terms of therapeutic effect, there is no accepted convergence for the minimum trial-level R^2^ value needed to verify a surrogacy endpoint, and different studies used different cut-off values (e.g., 0.60 [30, 31], 0.72 [44], and 0.75 [45, 46]). Irrespective of the chosen cutoff, according to the results of the present study, PFS is not acceptable as a valid OS surrogate. PD-L1 + HR_PFS_ and HR_OS_ exhibited an inverse correlation (R = -0.269) in our study, which could be explained by the delayed but durable treatment effect of immunotherapy [47–49]. Consequently, immunotherapy trials show a disassociation between short-term efficacy and long-term benefit. The lack of a strong PFS-OS correlation could be related to the traditional RECIST criteria. The clinical responses in five of the above 6 trials (CHECKMATE-648, KEYNOTE-590, ORIENT-15, ESCORT-1st, RATIONALE-306) was assessed only using RECIST V1.1, which was developed in the pre-immunotherapy era. In traditional RECIST criteria, disease progression is not adequately considered with immunotherapy in patients with atypical response patterns [50]. RECIST criteria specific for immunotherapy have been proposed; however, they have not yet been used in trials [12].

Continued immunotherapy beyond progression bring survival benefits in a substantial proportion of patients who appeared stable clinically and whose treating physicians determined that treatment was necessary beyond RECIST v1.1 progression. In our retrospective observational cohort, patients who continued PD-1/PD-L1 inhibitors treatment after progression had a longer PPS and lower annual risk of death than patients who did not. Following the first progression of their disease, patients obtained a long-term remissions to treatment and longer survival with continued immunotherapy in advanced melanoma [19], advanced hepatocellular carcinoma [20], and advanced gastric cancer [18]. Patients experience abnormal responses to inhibitors of PD-1/PD-L1 and an inflammatory reaction to immune checkpoint treatment might be confused with PD in radiographical assessments [43]; therefore, it might not be appropriate to cease treatment as soon as the tumor appears to have progressed if there are no adverse effects of the current treatment on the patient. The investigators’ decision to treat with PD-1/PD-L1 inhibitors beyond progression or to discontinue drugs should be based in part on patient's characteristics and clinical benefits [19, 20]. In two retrospective studies of nivolumab which have been conducted in patients with renal cell carcinoma of later period who have received TBP, new-onset bone lesions and insufficient quality of life scores were associated with a lower chance of receiving continued nivolumab therapy than those without these characteristics [42, 51]. Accordingly, treatment was unlikely to continue for patients with poor clinical characteristics.

Our study had some limitations. First, because the individual patient data could not be obtained from the 6 RCTs for our analyses, we only used a retrospective cohort data to evaluate PFS-OS relationships in patient-level, which ranks 0 as having a very poor validity in terms of statistical evaluation criteria for biomarker-surrogacy validity [32]. Second, Fig. 2 shows that there are too many censored cases both in PFS and OS because the follow-up period was not long enough. In addition, the RCTs did not have an adequate follow-up period, and the OS results have not been fixed. Although our follow-up time is consistent with the six RCTs, we have to admit that retrospective cohort studies are not as rigorous in design and standardized in follow-up system as prospective cohort studies. So, we need to improve the follow-up system and continue to follow up the patients in this study to obtain more specific OS results. Undeniably, at present, there are only 6 phase III RCTs of 1-line immunochemotherapy for advanced ESCC in the world, so based on the small number of comparisons in each analysis, the statistical power of the analysis is low. We need to collect more coming relevant RCTs data to verify our results. Fourth, without randomized data, it is not clear whether patients treated after progression would not have survived for so long in the absence of extended inhibitors of PD-1/PD-L1 treatment. Moreover, because the retrospective data are used in this study, the molecular pathological information of patients and the factors affecting the selection of patients for continuous treatment by investigators are not routinely collected. In the future, it will be necessary to conduct large prospective cohort studies to examine the prognosis of patients treated beyond progression, in which patients are randomly divided into a continuous treatment group and an observation only group.

In conclusion, PFS improvements of great magnitude are likely to have a positive impact on OS, according to our findings. However, in RCTs of anti-PD-1 agents + chemotherapy versus chemotherapy alone as 1-L treatment for advanced or metastatic ESCC, the current research provides only weak support for surrogating OS with PFS. In this case, PFS cannot completely replace OS as a reliable primary endpoint. OS should still be employed as the primary endpoint for prospective studies in the future to examine new immunotherapy drugs in advanced ESCC. Moreover, our analysis showed that patients experience a very durable survival benefit with combination immunochemotherapy, and treatment beyond first disease progression can achieve longer PPS and reduced annual risk of death with continuous anti-PD-1 therapy. The results of this study suggest that some patients with advanced ESCC might profit from continued PD-1/PD-L1 inhibitors to achieve further apparent clinical benefit.

## Supplementary Information

**Additional file 1:** **Supplement Methods.** Literature search strategy.

**Additional file 2:** **Supplement Table 1. **Summary of randomized controlled trials included in trial- and arm- level analyses in ITT population.

**Additional file 3:** **Supplement Table 2. **Summary of randomized controlled trials included in trial- and experimental arm-level analyses in PD-L1-positive population.

**Additional file 4:** **Supplement Figure S1. **Heterogeneityanalysis of the included studies. (A) Heterogeneity analysis of HR for PFS in the ITT population; (B) heterogeneity analysis of HR for OS in the ITTpopulation; (C) heterogeneity analysis of HR for PFS in the PD-L1+ population; (D)heterogeneity analysis of HR for OS in the PD-L1+ population. Abbreviations: ITT, intent-to-treat; PD-L1+, programmed death ligand-1 enriched.

**Additional file 5:** **Supplement Figure S2. **Publicationbias assessed with Egger’s test. (A) Publication bias of HR for PFS evaluated with Egger plot in the ITT population; (B) Publication bias of HR for OSevaluated with Egger plot in the ITT population; (C) Publication bias of HR for PFS evaluated with Egger plot in the PD-L1+ population; (D) Publication bias of HR for OS evaluated with Egger plot in the PD-L1+ population. Abbreviations: ITT, intent-to-treat; PD-L1+, programmed death ligand-1 enriched.

## Data Availability

The datasets generated and/or analysed during the current study are not publicly available due to privacy or ethical restrictions but are available from the corresponding author on reasonable request.
