# Supplementary material for: Identification on surrogating overall survival with progression-free survival of first-line immunochemotherapy in advanced esophageal squamous cell carcinoma—an exploration of surrogate endpoint
Source: BMC Cancer. 2023 Feb 10;23:145. doi: 10.1186/s12885-023-10613-y (PMC9921746; doi:10.1186/s12885-023-10613-y)
Supplement: Supplementary file 1 — Additional file 1: Supplement Methods. Literature search strategy. [file 12885_2023_10613_MOESM1_ESM.docx]

**Supplement Methods**

**Literature search strategy**

1. **Pubmed**

#1 **((randomized controlled trial [pt]) OR (controlled clinical trial [pt]) OR (randomized [tiab]) OR (placebo [tiab]) OR (clinical trials as topic [mesh: noexp]) OR (randomly [tiab]) OR (trial [ti]))**

#2 **animals [mh] NOT humans [mh]**

#3 **((((((((((((((((((((((((((((((((((((((((((((((((((((((((((Nivolumab) OR (Opdivo)) OR (BMS-936558)) OR (MDX1106)) OR (ONO-4538)) OR (BMS-936559)) OR (MDX1105)) OR (Pembrolizumab)) OR (Lambrolizumab)) OR (Keytruda)) OR (MK-3475)) OR (Atezolizumab)) OR (MPDL3280)) OR (MPDL3280A)) OR (RG7446)) OR (Tecentriq)) OR (Avelumab)) OR (MSB0010718C)) OR (Durvalumab)) OR (MEDI4736)) OR (cemiplimab)) OR (REGN2810)) OR (PD-1 inhibitor)) OR (Programmed death 1 inhibitor)) OR (Anti-PD-1)) OR (Anti-Programmed Cell Death 1)) OR (PD-L1 inhibitor)) OR (Programmed death ligand 1 inhibitor)) OR (Anti-PD-L1)) OR (Anti-Programmed Cell Death Ligand-1)) OR (Checkpoint Inhibitor)) OR (Checkpoint blockade)) OR (Programmed Cell Death 1 Receptor [mesh])) OR (CTLA4 protein, human)) OR (anti CTLA4)) OR (anti CTLA-4)) OR (cytotoxic T-lymphocyte-associated antigen 4)) OR (Cytotoxic T lymphocyte antigen 4)) OR (CTLA-4)) OR (Cytotoxic T-lymphocyte protein 4)) OR (anti-CTLA4 antibodies)) OR (Ipilimumab)) OR (MDX-010)) OR (MDX-101)) OR (BMS-734016)) OR (Yervoy)) OR (Antigens, CD/immunology* [mesh])) OR (CTLA-4 Antigen [mesh])) OR (Atezolizumab)) OR (Tecentriq)) OR (Imfinzi)) OR (Durvalumab)) OR (Tislelizumab)) OR (Camrelizumab)) OR (Sintilimab)) OR (Toripalimab)) OR (cemiplimab)) OR (Avelumab)) OR (Lambrolizumab)**

#4 **((esophageal[Title/Abstract]) OR (oesophageal[Title/Abstract])) OR (Gastroesophageal[Title/Abstract])**

#5 **(((#1) NOT (#2)) AND (#3)) AND (#4)**

**B.Embase**

**#1 'esophagus tumor'/exp OR 'esophagus cancer'/exp OR 'esophagus carcinoma'/exp**

**#2 'crossover procedure':de OR 'double-blind procedure':de OR 'randomized controlled trial':de OR 'single-blind procedure':de OR random*:de,ab,ti OR factorial*:de,ab,ti OR crossover*:de,ab,ti OR ((cross NEXT/1 over*):de,ab,ti) OR placebo*:de,ab,ti OR ((doubl* NEAR/1 blind*):de,ab,ti) OR ((singl* NEAR/1 blind*):de,ab,ti) OR assign*:de,ab,ti OR allocat*:de,ab,ti OR volunteer*:de,ab,ti**

**#3 nivolumab OR opdivo OR pembrolizumab OR keytruda OR atezolizumab OR tecentriq OR imfinzi OR durvalumab OR tislelizumab OR camrelizumab OR sintilimab OR toripalimab OR cemiplimab OR avelumab OR lambrolizumab OR 'pd-1 inhibitor' OR 'programmed death 1 inhibitor' OR 'anti-pd-1' OR 'anti-programmed cell death 1' OR 'pd-l1 inhibitor' OR 'programmed death ligand 1 inhibitor' OR 'anti-pd-l1' OR 'anti-programmed cell death ligand-1' OR 'checkpoint inhibitor' OR 'checkpoint blockade' OR 'pd-1' OR 'pd-l1' OR immuno*:de,ab,ti OR 'programmed cell death 1 receptor'/de OR 'ctla4 protein' OR 'anti ctla4' OR 'anti ctla-4' OR 'cytotoxic t-lymphocyte-associated antigen 4' OR 'cytotoxic t lymphocyte antigen 4' OR 'ctla-4' OR 'cytotoxic t-lymphocyte protein 4' OR 'anti-ctla4 antibodies' OR 'ctla-4 antigen' OR 'ipilimumab'**

**#1 AND #2 AND #3 AND [english]/lim**

**C.Cochrane**

#1 (esophageal):ti,ab,kw OR (oesophageal):ti,ab,kw OR (gastroesophageal):ti,ab,kw (Word variations have been searched)

#2 (cancer):ti,ab,kw OR (carcinoma):ti,ab,kw

#3 (Nivolumab OR Opdivo OR Pembrolizumab OR Keytruda OR Atezolizumab OR Tecentriq OR Imfinzi OR Durvalumab OR Tislelizumab OR Camrelizumab OR Sintilimab OR Toripalimab OR cemiplimab OR Avelumab OR Lambrolizumab OR (PD-1 inhibitor) OR (Programmed death 1 inhibitor) OR (Anti-PD-1) OR (Anti-Programmed Cell Death 1) OR (PD-L1 inhibitor) OR (Programmed death ligand 1 inhibitor) OR (Anti-PD-L1) OR (Anti-Programmed Cell Death Ligand-1) OR (Checkpoint Inhibitor) OR (Checkpoint blockade) OR (PD-1) OR (pd-l1) OR (immuno*) OR (Programmed Cell Death 1 Receptor) OR (CTLA4 protein) OR (anti CTLA4) OR (anti CTLA-4) OR (cytotoxic T-lymphocyte-associated antigen 4) OR (Cytotoxic T lymphocyte antigen 4) OR (CTLA-4) OR (Cytotoxic T-lymphocyte protein 4) OR (anti-CTLA4 antibodies) OR (Ipilimumab) OR (MDX-010) OR (MDX-101) OR (BMS-734016) OR (Yervoy) OR (CTLA-4 Antigen)):ti,ab,kw (Word variations have been searched)

#4 MeSH descriptor: [B7-H1 Antigen] explode all trees

#5 MeSH descriptor: [CTLA-4 Antigen] explode all trees

#6 MeSH descriptor: [Programmed Cell Death 1 Receptor] explode all trees

#7 #1 AND #2 AND (#3 OR #4 OR #5 OR #6)
