# Supplementary material for: Identification on surrogating overall survival with progression-free survival of first-line immunochemotherapy in advanced esophageal squamous cell carcinoma—an exploration of surrogate endpoint
Source: BMC Cancer. 2023 Feb 10;23:145. doi: 10.1186/s12885-023-10613-y (PMC9921746; doi:10.1186/s12885-023-10613-y)
Supplement: Supplementary file 2 — Additional file 2: Supplement Table 1. Summary of randomized controlled trials included in trial- and arm- level analyses in ITT population. [file 12885_2023_10613_MOESM2_ESM.docx]

| **Supplement Table 1. Summary of randomized controlled trials included in trial- and arm- level analyses in ITT population.** | | | | | | | | | | |
| --- | --- | --- | --- | --- | --- | --- | --- | --- | --- | --- |
| Trial name | Eligibility | Phase | Treatment line | Arm | Treatment  Protocol | Participants, No. | Progression free survival | | Overall survival | |
|  |  |  |  |  |  |  | mPFS(95%CI) , mo | HR(95% CI) | mOS(95%CI), mo | HR (95% CI) |
| Sun 2021, KEYNOTE-590 ,  NCT03189719[24] | advanced or metastatic ESCC | III | 1 | PD-1 plus chemotherapy  group | Pembrolizumab (200mg Q3W), 5-FU (800mg/m2 Q3W), cisplatin (80mg/m2 Q3W) | 274 | 6.3 (6.2-6.9) | 0.65 (0.54-0.78) | 12.6 (10.2-14.3) | 0.72 (0.60-0.88) |
|  |  |  |  | Placebo plus  chemotherapy  group | placebo (200mg Q3W), 5-FU (800mg/m2 Q3W), cisplatin (80mg/m2 Q3W) | 274 | 5.8 (5.0-6.1) |  | 9.8 (8.6-11.1) |  |
| Doki 2022, CheckMate-648, NCT03143153[6] | advanced or metastatic ESCC | III | 1 | PD-1 plus chemotherapy  group | Nivolumab (240mg Q2W), 5-FU (800mg/m2 Q4W), cisplatin (80mg/m2 Q4W) | 321 | 5.8 (5.6-7.0) | 0.81  (98.5% CI, 0.64-1.04) | 13.2 (11.1-15.7) | 0.74 (0.61-0.89) |
|  |  |  |  | Placebo plus  chemotherapy  group | placebo (240mg Q2W), 5-FU (800mg/m2 Q4W), cisplatin (80mg/m2 Q4W) | 324 | 5.6 (4.3-5.9) |  | 10.7 (9.4-11.9) |  |
| Luo 2021, ESCORT-1st ,  NCT03691090[21] | advanced or metastatic ESCC | III | 1 | PD-1 plus chemotherapy  group | Camrelizumab (200mg, Q3W), paclitaxel (175mg/m2 Q3W), cisplatin (75mg/m2 Q3W) | 298 | 6.9 (5.8-7.4) | 0.56 (0.46-0.68) | 15.3 (12.8-17.3) | 0.70 (0.56-0.88) |
|  |  |  |  | Placebo plus  chemotherapy  group | placebo (200mg, Q3W), paclitaxel (175mg/m2 Q3W), cisplatin (75mg/m2 Q3W), | 298 | 5.6 (5.5-5.7) |  | 12.0 (11.0-13.3) |  |
| Wang 2022, JUPITER-06,  NCT03829969[5] | advanced or metastatic ESCC | III | 1 | PD-1 plus chemotherapy  group | Toripalimab (240mg Q3W), paclitaxel (175mg/m2 Q3W), cisplatin (75mg/m2 Q3W) | 257 | 5.7 (5.6-7.0) | 0.58 (0.46-0.74) | 17.0 (14.0-NE) | 0.58 (0.43-0.78) |
|  |  |  |  | Placebo plus  chemotherapy  group | placebo (240mg Q3W), paclitaxel (175mg/m2 Q3W), cisplatin (75mg/m2 Q3W) | 257 | 5.5 (5.2-5.6) |  | 11.0 (10.4-12.6) |  |
| Lu 2022, ORIENT-15,  NCT03748134[23] | advanced or metastatic ESCC | III | 1 | PD-1 plus chemotherapy  group | Sintilimab (200mg Q3W), cisplatin (75mg/m2 Q3W) plus paclitaxel (175mg/m2 Q3W) or 5-FU (800mg/m2 Q3W) | 327 | 7.2 (7.0-9.6) | 0.56 (0.46-0.68) | 16.7 (14.8-21.7) | 0.63 (0.51-0.78) |
|  |  |  |  | Placebo plus  chemotherapy  group | placebo (200mg Q3W), cisplatin (75mg/m2 Q3W) plus paclitaxel (175mg/m2 Q3W) or 5-FU (800mg/m2 Q3W) | 332 | 5.7 (5.5-6.8) |  | 12.5 (11.0-14.5) |  |
| Yoon 2022, RATIONALE-306,  NCT03783442[25] | advanced or metastatic ESCC | III | 1 | PD-1 plus chemotherapy  group | Tislelizumab (200mg Q3W), platinum (Cisplatin 60–80 mg/m2 or oxaliplatin 130 mg/m2 Q3W) plus paclitaxel (175mg/m2 Q3W) or fluoropyrimidine (5-FU 750-800mg/m2 Q3W or capecitabine 1000 mg/m2 orally BID on Days 1–14) | 326 | 7.3 (6.9-8.3) | 0.62 (0.52-0.75) | 17.2 (15.8-20.1) | 0.66 (0.54-0.80) |
|  |  |  |  | Placebo plus  chemotherapy  group | placebo (200mg Q3W), platinum (Cisplatin 60–80 mg/m2 or oxaliplatin 130 mg/m2 Q3W) plus paclitaxel (175mg/m2 Q3W) or fluoropyrimidine (5-FU 750-800mg/m2 Q3W or capecitabine 1000 mg/m2 orally BID on Days 1–14) | 323 | 5.6 (4.9-6.0) |  | 10.6 (9.3-12.1) |  |
| Abbreviations: ESCC, esophageal squamous cell carcinoma; ITT, intention-to-treat; PD-1, programmed cell death-1 (PD-1) inhibitors; mOS, median overall survival; mPFS, median progression-free survival; mo, months; HR, hazard ratio; CI, confidence interval. | | | | | | | | | | |
