# Supplementary material for: Identification on surrogating overall survival with progression-free survival of first-line immunochemotherapy in advanced esophageal squamous cell carcinoma—an exploration of surrogate endpoint
Source: BMC Cancer. 2023 Feb 10;23:145. doi: 10.1186/s12885-023-10613-y (PMC9921746; doi:10.1186/s12885-023-10613-y)
Supplement: Supplementary file 3 — Additional file 3: Supplement Table 2. Summary of randomized controlled trials included in trial- and experimental arm-level analyses in PD-L1-positive population. [file 12885_2023_10613_MOESM3_ESM.docx]

| **Supplement Table 2. Summary of randomized controlled trials included in trial- and experimental arm-level analyses in PD-L1-positive population.** | | | | | | | | |
| --- | --- | --- | --- | --- | --- | --- | --- | --- |
| Trial | Arm | Detection of PD-L1 tumor expression | PD-L1 expression | Participants, No. | Progression free survival | | Overall survival | |
|  |  |  |  |  | mPFS(95%CI), mo | HR (95%CI) | mOS(95%CI), mo | HR (95%CI) |
| Sun 2021, KEYNOTE-590 | Pembrolizumab plus chemotherapy  group | PD-L1 IHC 22C3 assay (Agilent Technologies, Carpinteria, CA, USA) | CPS≥10 | 143 | 7.3 (6.2-8.2) | 0.53 (0.40-0.69) | 13.9 (11.1-17.7) | 0.57 (0.43-0.75) |
|  | Placebo plus  chemotherapy  group |  |  | 143 | 5.4 (4.2-6.0) |  | 8.8 (7.8-10.5) |  |
| Doki 2022, CHECKMATE-648 | Nivolumab plus chemotherapy  group | PD-L1 IHC 28-8 assay  (Dako, an Agilent Technologies, Inc. company, Santa Clara, CA, USA) | TPS≥1% | 158 | 6.9 (5.7–8.3) | 0.65  (98.5% CI, 0.46–0.92) | 15.4 (11.9–19.5) | 0.54 (0.42–0.71) |
|  | Placebo plus  chemotherapy  group |  |  | 157 | 4.4 (2.9–5.8) |  | 9.1 (7.7–10.0) |  |
| Luo 2021, ESCORT-1st | Camrelizumab plus chemotherapy  group | PD-L1 IHC 6E8 assay  (Abcam) | TPS≥1% | 166 | 6.9 (5.7-7.8) | 0.51 (0.39-0.67) | 15.3 (12.4-NR) | 0.59 (0.43-0.80) |
|  | Placebo plus  chemotherapy  group |  |  | 163 | 5.6 (5.4-5.7) |  | 11.5 (10.3-13.3) |  |
| Wang 2022, JUPITER-06 | Toripalimab plus chemotherapy  group | PD-L1 IHC JS311 assay  (MEDx Translational Medicine Co. Ltd.) | CPS≥1 | 201 | 5.7 (5.6-7.0) | 0.58 (0.44-0.75) | 15.2 (13.2-NE) | 0.61 (0.44-0.87) |
|  | Placebo plus  chemotherapy  group |  |  | 200 | 5.5 (5.1-5.6) |  | 10.9 (10.0-12.6) |  |
| Lu 2022, ORIENT-15 | Sintilimab plus chemotherapy  group | PD-L1 IHC 22C3 assay (Agilent Technologies, Carpinteria, CA, USA) | CPS≥10 | 188 | 8.3 (6.9- 12.4) | 0.58 (0.45-0.75) | 17.2 (15.5 to NC) | 0.64 (0.48-0.85) |
|  | Placebo plus  chemotherapy  group |  |  | 193 | 6.4 (5.5 to 6.9) |  | 13.6 (11.3 to 15.7) |  |
| Abbreviations: ESCC, esophageal squamous cell carcinoma; PD-1, programmed cell death-1 (PD-1) inhibitors; PD-L1, programmed death ligand-1; mOS, median overall survival; mPFS, median progression-free survival;  mo, months; HR, hazard ratio; CI, confidence interval. | | | | | | | | |
