# Supplementary figures and images for: Identification on surrogating overall survival with progression-free survival of first-line immunochemotherapy in advanced esophageal squamous cell carcinoma—an exploration of surrogate endpoint
Source: BMC Cancer. 2023 Feb 10;23:145. doi: 10.1186/s12885-023-10613-y (PMC9921746; doi:10.1186/s12885-023-10613-y)

(B)

Study

%

ID

HR (95% CI)

Weight

Sun 2021

0.72 (0.60, 0.88)

20.32

Doki 2022

0.74 (0.61, 0.89)

20.89

Luo 2021

0.70 (0.56, 0.88)

14.59

Wang 2022

0.58 (0.43, 0.78)

8.40

Lu 2022

0.63 (0.51, 0.78)

16.51

Yoon 2022

0.66 (0.54, 0.80)

19.29

Overall (I-squared = 0.0%, p = 0.722)

0.68 (0.62, 0.74)

100.00

.43

1

2.33

## Study

ID

**Weight**

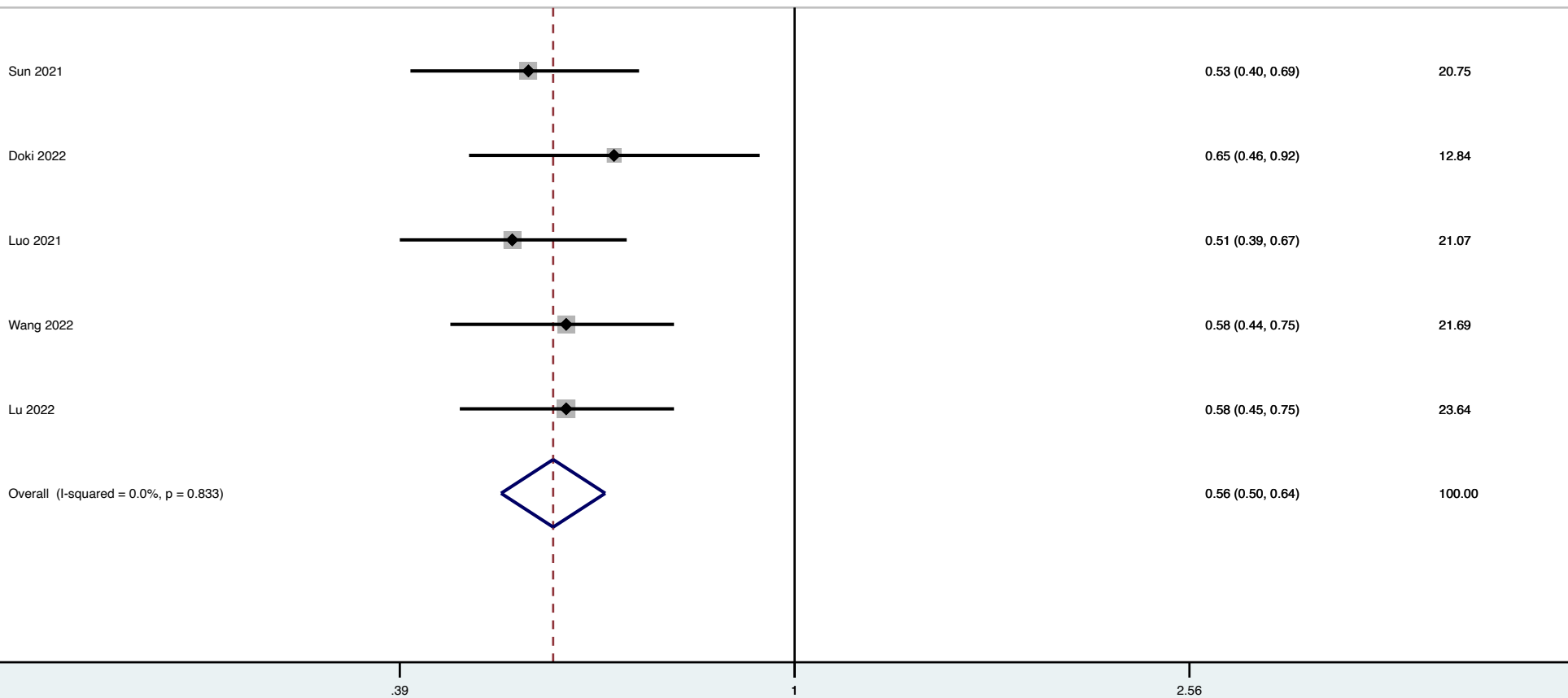

(D)

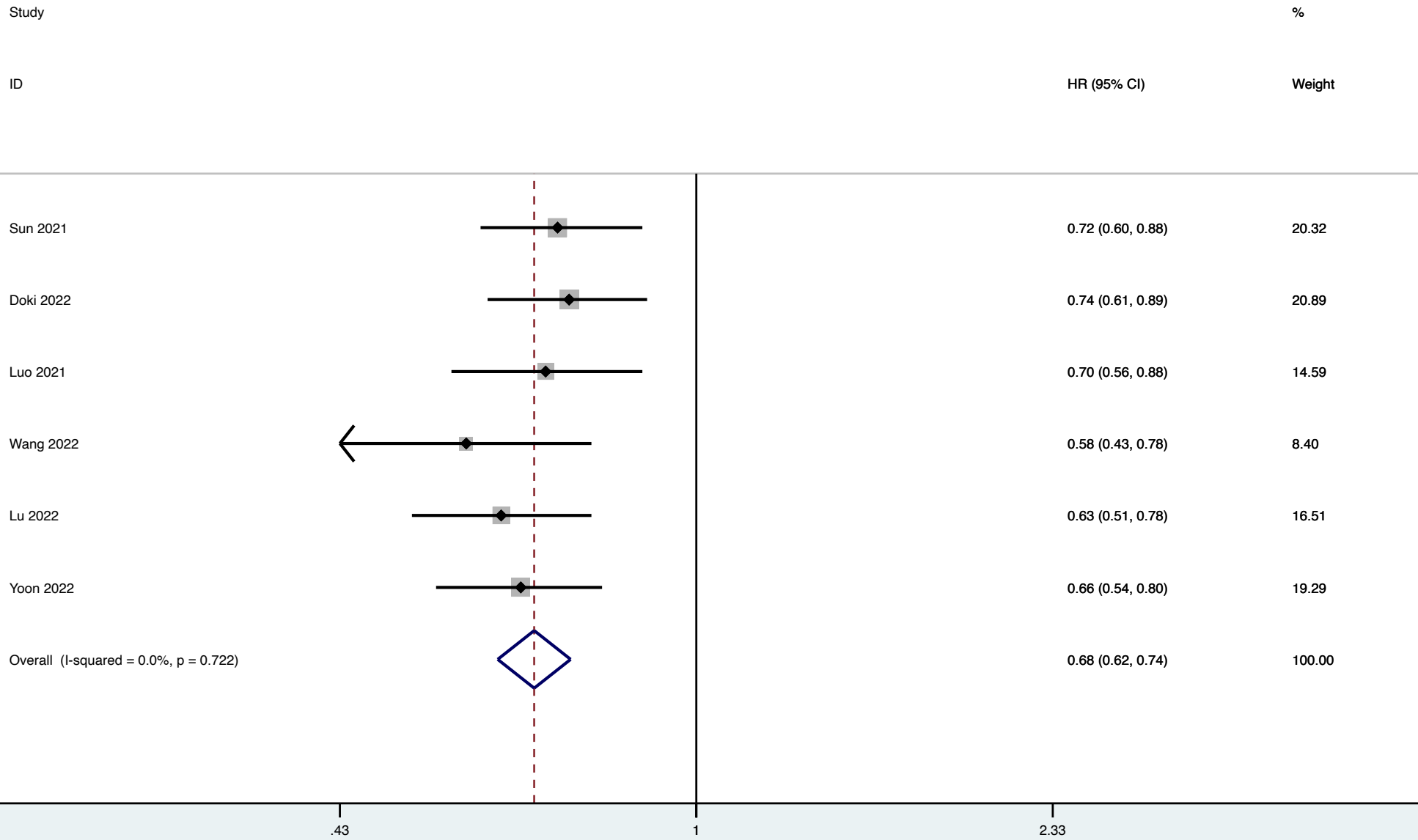

Supplement: Supplementary file 4 — Additional file 4: Supplement Figure S1. Heterogeneityanalysis of the included studies. (A) Heterogeneity analysis of HR for PFS in the ITT population; (B) heterogeneity analysis of HR for OS in the ITTpopulation; (C) heterogeneity analysis of HR for PFS in the PD-L1+ population; (D)heterogeneity analysis of HR for OS in the PD-L1+ population. Abbreviations: ITT, intent-to-treat; PD-L1+, programmed death ligand-1 enriched. [file 12885_2023_10613_MOESM4_ESM.pdf]
