# Supplementary material for: Identification on surrogating overall survival with progression-free survival of first-line immunochemotherapy in advanced esophageal squamous cell carcinoma—an exploration of surrogate endpoint
Source: BMC Cancer. 2023 Feb 10;23:145. doi: 10.1186/s12885-023-10613-y (PMC9921746; doi:10.1186/s12885-023-10613-y)
Supplement: Supplementary file 5 — Additional file 5: Supplement Figure S2. Publicationbias assessed with Egger’s test. (A) Publication bias of HR for PFS evaluated with Egger plot in the ITT population; (B) Publication bias of HR for OSevaluated with Egger plot in the ITT population; (C) Publication bias of HR for PFS evaluated with Egger plot in the PD-L1+ population; (D) Publication bias of HR for OS evaluated with Egger plot in the PD-L1+ population. Abbreviations: ITT, intent-to-treat; PD-L1+, programmed death ligand-1 enriched. [file 12885_2023_10613_MOESM5_ESM.pdf]

(A)

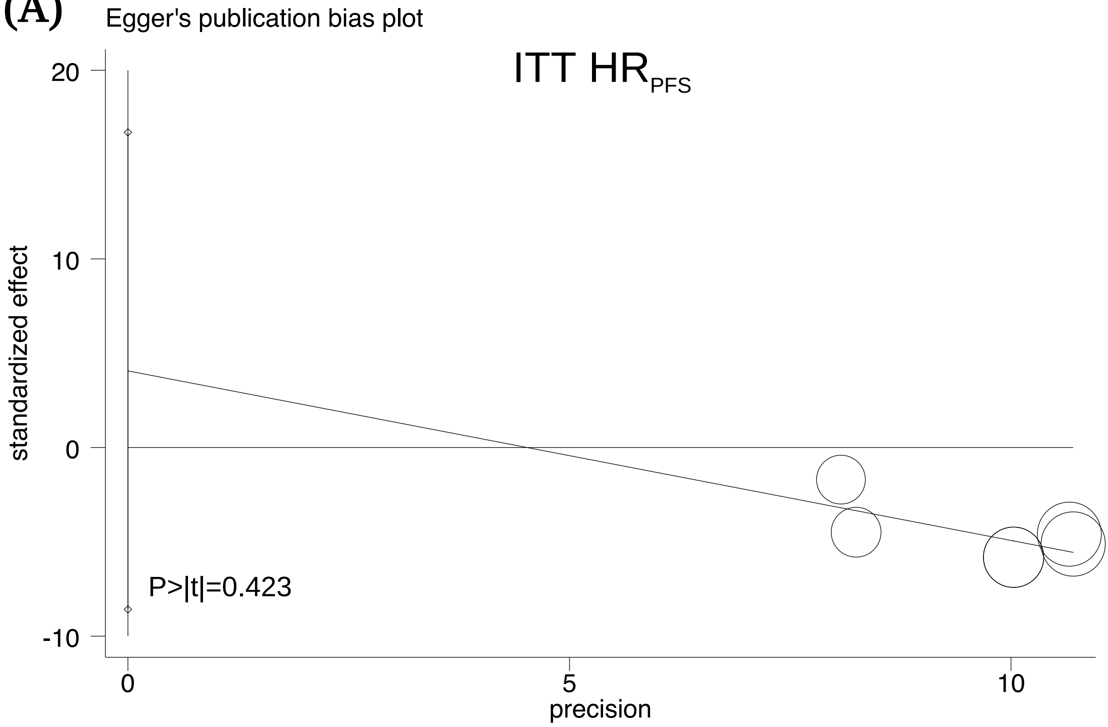

Egger's publication bias plot
ITT HR PFS
20
10
0
-10
standardized effect
0
5
10
precision
P>|t|=0.423

(B)

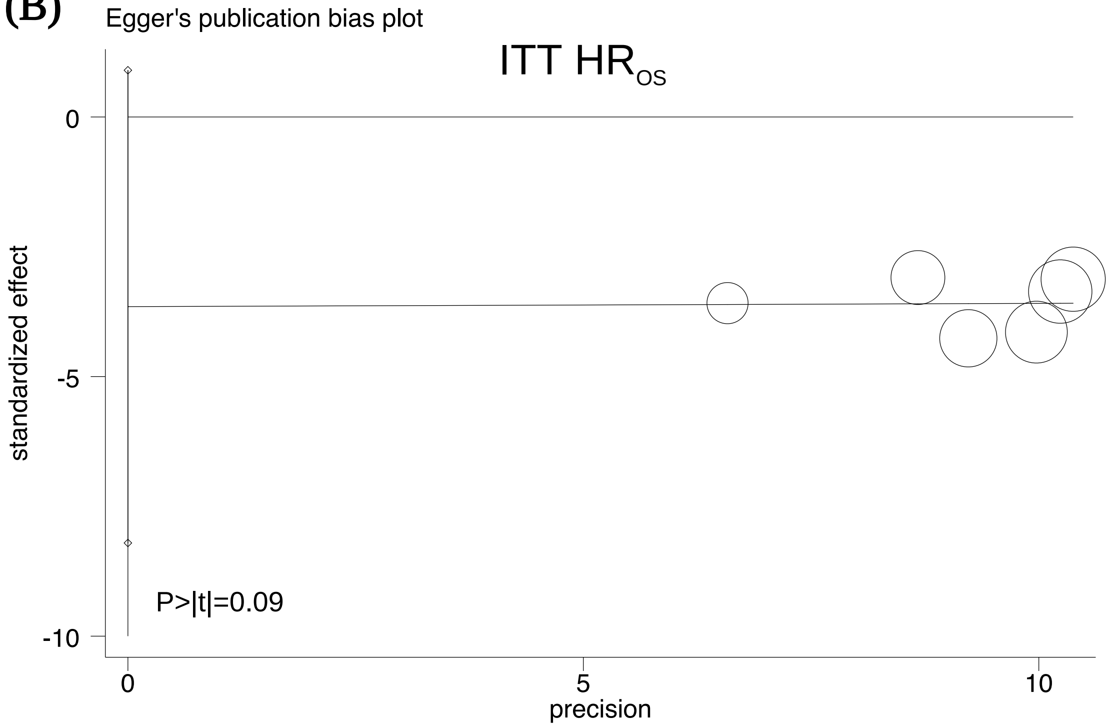
Egger's publication bias plot
ITT HR_OS
0
-5
-10
standardized effect
P>|t|=0.09
0
5
10
precision

(C)

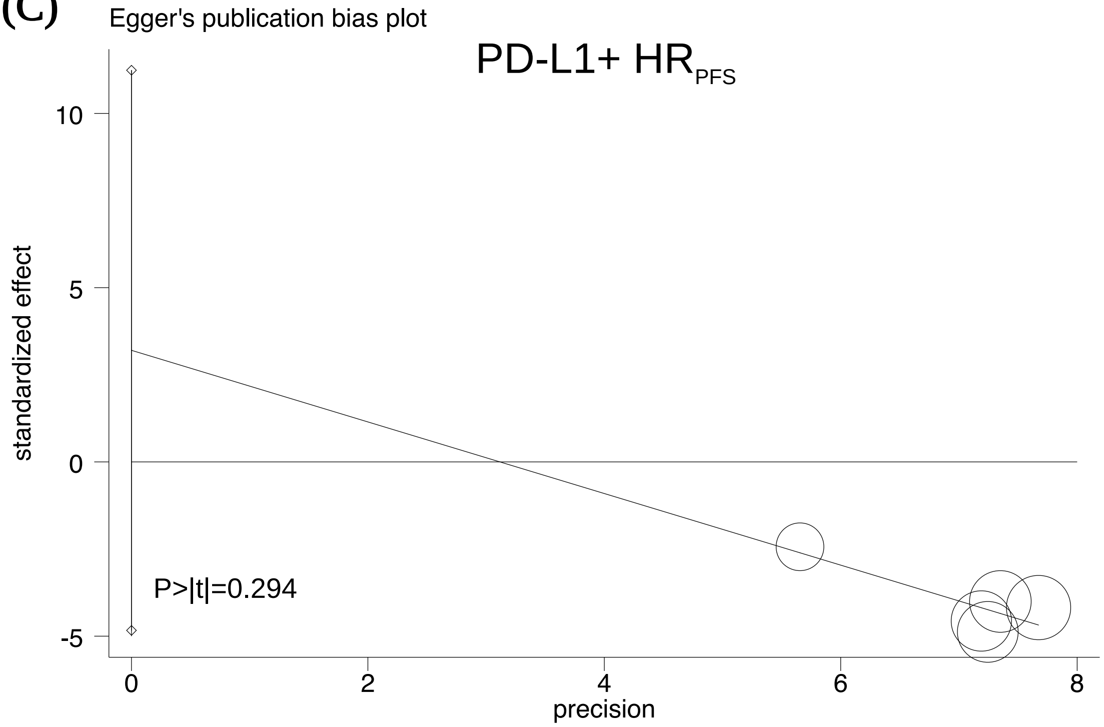
Egger's publication bias plot
PD-L1+ HR PFS
standardized effect
10
5
0
-5
P>|t|=0.294
0
2
4
6
8
precision

(D)

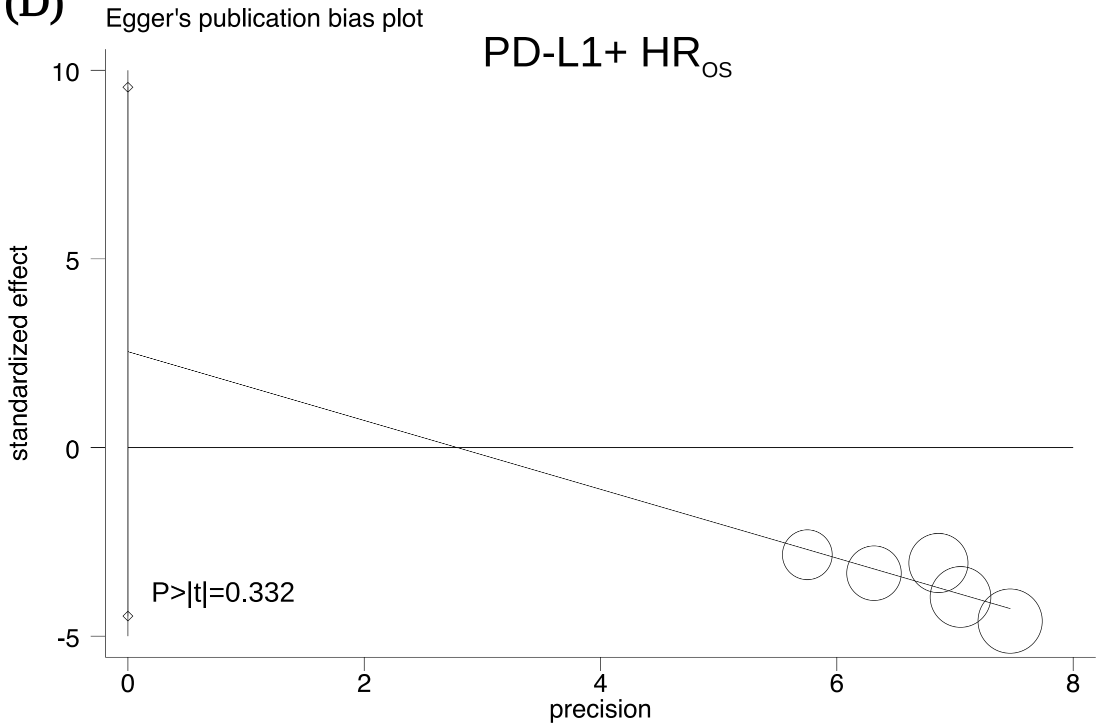

Egger's publication bias plot
PD-L1+ HR_OS
10
5
0
-5
standardized effect
P>|t|=0.332
0
2
4
6
8
precision
